# Sex classification of 3D skull images using deep neural networks

**DOI:** 10.1038/s41598-024-61879-6

**Published:** 2024-06-14

**Authors:** Lake Noel, Shelby Chun Fat, Jason L. Causey, Wei Dong, Jonathan Stubblefield, Kathryn Szymanski, Jui-Hsuan Chang, Paul Zhiping Wang, Jason H. Moore, Edward Ray, Xiuzhen Huang

**Affiliations:** 1 Department of Computational Biomedicine, Cedars Sinai Medical Center, Los Angeles, CA USA; 2 Department of Surgery, Cedars Sinai Medical Center, Los Angeles, CA USA; 3 Ann Arbor Algorithms, Ann Arbor, MI USA; 4 Center for No-Boundary Thinking (CNBT), Arkansas State University, Jonesboro, AR USA; 5 Department of Computer Science, Arkansas State University, Jonesboro, AR USA; 6 School of Medicine, Creighton University, Omaha, NE USA

**Keywords:** Plastic surgery, 3D medical imaging, Deep learning, Medical research, Medical imaging

## Abstract

Determining the fundamental characteristics that define a face as "feminine" or "masculine" has long fascinated anatomists and plastic surgeons, particularly those involved in aesthetic and gender-affirming surgery. Previous studies in this area have relied on manual measurements, comparative anatomy, and heuristic landmark-based feature extraction. In this study, we collected retrospectively at Cedars Sinai Medical Center (CSMC) a dataset of 98 skull samples, which is the first dataset of this kind of 3D medical imaging. We then evaluated the accuracy of multiple deep learning neural network architectures on sex classification with this dataset. Specifically, we evaluated methods representing three different 3D data modeling approaches: Resnet3D, PointNet++, and MeshNet. Despite the limited number of imaging samples, our testing results show that all three approaches achieve AUC scores above 0.9 after convergence. PointNet++ exhibits the highest accuracy, while MeshNet has the lowest. Our findings suggest that accuracy is not solely dependent on the sparsity of data representation but also on the architecture design, with MeshNet's lower accuracy likely due to the lack of a hierarchical structure for progressive data abstraction. Furthermore, we studied a problem related to sex determination, which is the analysis of the various morphological features that affect sex classification. We proposed and developed a new method based on morphological gradients to visualize features that influence model decision making. The method based on morphological gradients is an alternative to the standard saliency map, and the new method provides better visualization of feature importance. Our study is the first to develop and evaluate deep learning models for analyzing 3D facial skull images to identify imaging feature differences between individuals assigned male or female at birth. These findings may be useful for planning and evaluating craniofacial surgery, particularly gender-affirming procedures, such as facial feminization surgery.

## Introduction

Gender-affirming surgery (GAS), including facial feminization surgery (FFS), has been offered at increasing rates since the 1980s, with strong evidence of resulting psychological and quality-of-life improvement among individuals with gender dysphoria. In this text, we use the terms “male”, “masculine”, “female”, and “feminine” to refer to sexually dimorphic phenotypic traits and not gender identity (shaped by social and cultural circumstances). Understanding what makes a face fundamentally “feminine” or “masculine” is a question that has intrigued anatomists and plastic surgeons alike for generations^1–3^. Prior research in this field has relied upon manual measurements and comparative anatomy biased toward specific “ideal” facial dimensions without sufficient consideration of racial and geographic variations or patient-reported outcomes^4,5^. Previous machine learning approaches for sex determination have used methods based on heuristic landmark-based feature extraction.

In this study, we retrospectively collected a dataset of 98 skull image samples, the first dataset of its kind of 3D medical imaging. First, we assessed the accuracy of multiple deep neural network architectures on sex classification using this dataset. Specifically, we evaluated methods representing three different approaches for 3D data modeling: Resnet3D^6^, PointNet++^7^, and MeshNet^8^. Resnet3D utilizes dense 3D volumes while PointNet++ and MeshNet utilize sparse representation based on meshes, with PointNet++ using vertices while MeshNet uses faces. Our results show that PointNet++ achieves the best accuracy out of the three and MeshNet has the lowest accuracy. Therefore, accuracy is not merely determined by sparsity of data representation but depends on more details of the architecture design. The low accuracy of MeshNet is likely due to the lack of a hierarchical structure for progressive data abstraction.

Next, we studied the problem of sex determination, through the analysis of the various morphological features that contribute to sexual dimorphism. Facial feminization surgery alters the shape of the facial skeleton so that it is more representative of the individual’s gender identity. Previous studies on dimorphic traits have mainly relied upon population statistics. A machine learning model provides a convenient way to identify such features on a sample skull and to suggest potential modifications to achieve the phenotype that aligns with that individual’s gender identity. The feature importance to a deep learning model is typically visualized using a saliency map. However, the standard saliency map faces a challenge in visualization of meaningful information about 3D skulls, especially features on the frontal surface. We developed a new method based on morphological gradients, an alternative to the standard saliency map that provides better visualization of feature importance.

Our contributions in this paper include evaluating three different neural network models for sex classification of 3D skull images, developing an open-source deep learning framework for efficient processing and augmentation of 3D mesh data, and proposing a new method based on morphological gradients to visualize features that influence model decision-making. These contributions could potentially aid in planning and evaluating the results of facial feminization surgery (FFS).

Our work's interdisciplinary nature bridges the gap between the analytical precision of AI deep learning techniques and the nuanced requirements of facial plastic and craniofacial surgery, particularly in the context of gender-affirming procedures like FFS. By providing a deeper understanding of sexual dimorphism in cranial morphology and introducing novel methods for its analysis, our study not only has the potential to enhance surgical outcomes but also offers valuable insights for fields such as forensic anthropology, where sex determination plays a crucial role.

## Materials and methods

### Data acquisition

This study was conducted with approval from the Institutional Review Board at Cedars Sinai Medical Center; a waiver of informed consent was granted by the Institutional Review Board Committee due to the retrospective nature of the study. All methods were performed in accordance with relevant guidelines and regulations. To construct the dataset, we selected radiographic studies from all facial computed tomograms (CTs) obtained between January 1, 2015 through January 1, 2023, stored in our institutional picture archiving and communication system (PACS). All facial CT scans were acquired with a Discovery CT750 HD high-definition detector (GE Healthcare, Chicago, Illinois, USA) with the following parameters: 0.625 mm thickness, 0.312 mm reconstruction interval, 512 × 512 image matrix.

To avoid bias, all sequential exams meeting criteria were collected until a sufficient and relatively equal number of male and female subjects were included. Inclusion criteria required participants to be over 18 years of age, without evidence of facial trauma or congenital deformity. Patients with hardware or displaced fractures in their facial skeleton, incomplete radiologic studies, or who opted out of inclusion in research studies were excluded. Prior to de-identification and export, we tabulated sex assigned at birth, age, and racial identity as well as the relevant medical and surgical history. Participants with medical histories that could potentially affect their morphology and appearance (e.g., use of exogenous sex hormones) were excluded from the study.

The image files were exported in DICOM (Digital Imaging and Communications in Medicine) by one team member (not involved in the analysis) from the PACS into Vitrea^®^ advanced visualization software (Canon Medical, Minnetonka, MN). The files were de-identified in Vitrea^®^ and assigned a tracking number, then exported in STL (standard triangle language) format for neural network processing.

**Figure 1:**
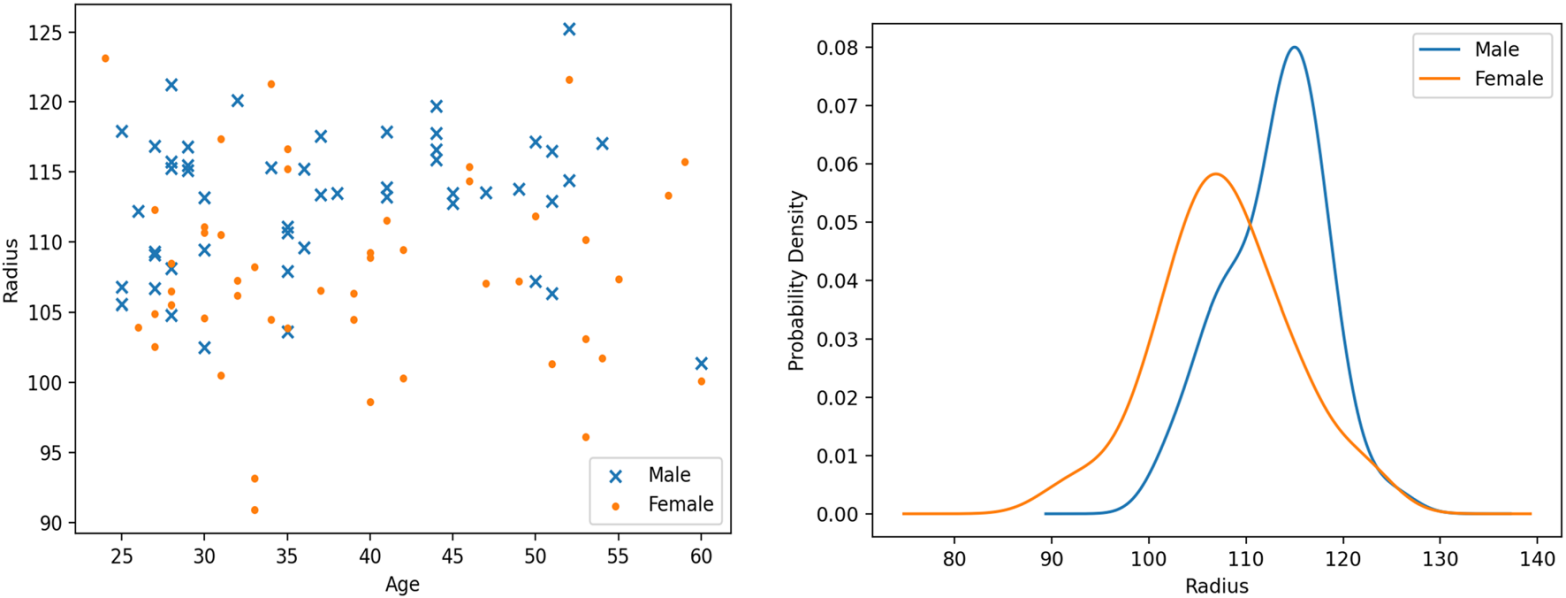
(Left) Age versus radius of the bounding sphere. (Right) Probability distribution of the radius.

The dataset included a total of 98 skulls, 50 male and 48 female, with ages ranging from 24 to 60 years. The self-identified racial makeup was 56 White, 19 Black, 6 Asian, and 17 Other. Figure 1 shows the skull size and age distributions. It is evident from both plots that male skulls are generally larger than female skulls, and there is no visible impact of age on skull size. As size is a discriminating feature, size-normalizing the skull to a unit sphere typically lowers model accuracy.

For the 3D tomographic data obtained via CT, the raw 3D image data is large and inefficient to process. For CT data, bone significantly attenuates x-rays and thus has a high Hounsfield unit (HU) value, making it easy to differentiate from other tissues. When only the bone structure is of interest, the relevant bony regions can first be extracted as a binary mask and then converted into a 3D mesh. This is well handled with today’s computer graphics software and hardware.

### Overview of 3D neural networks

This subsection provides an overview of the three neural network approaches we evaluated in this study, namely, 3D Convolutional Neural Network (CNN) (represented by Resnet3D), PointNet++, and MeshNet.

#### 3D CNN

3D CNN is an extension of 2D CNN that has achieved significant success in image recognition tasks. In this study, we evaluated the 3D version of Resnet, a popular off-the-shelf model. However, the 3D version of CNN has practical challenges that need to be addressed. One major challenge is that increasing the number of dimensions from two to three substantially increases data size for the same linear size and resolution. The data size of a single volume of 256^3^ is the same as a batch of 256 images of 256^2^, which is very close to the capacity limit of today’s high-end graphics cards. The size of data poses a limit on the input data resolution as well as the maximal parametric capacity of the neural network.

Yet another subtle issue that arises from the intrinsic property of 3D space is the ratio between boundary and internal data units. The very basic idea of convolution is to match data against the pattern represented by the convolution kernel, and such pattern is best captured when its signature is close to the center of the kernel. For a 2D kernel of 3 × 3, 8 out of 9 pixels are on the boundary, so the ratio is 88.9%. However, for a 3D kernel of 3 × 3 × 3, the ratio is 96.3%. Larger kernel boundaries mean that a pattern is more difficult to be captured, since data tends to change on the boundary.

Finally, loading and augmentation of 3D volumes, especially random rotation, can be time-consuming and can easily become a computational bottleneck if not handled properly. In this study, we apply augmentation in the mesh format and then voxelize the mesh using a GPU, as detailed in the data pipeline subsection.

The other two approaches described below work with sparse (i.e., mesh) representation. Some data like CT of soft tissues are intrinsically volumetric and cannot be easily converted into a sparse format without substantial loss of information, and in such cases, only 3D CNNs are applicable. However, our case benefits from the fact that there is a clear decision boundary between bones and soft tissues and the data can be converted to mesh without much loss of information.

#### PointNet++ and MeshNet

Both PointNet++ and MeshNet are based on sparse representations, and we summarize them under the same general framework. Please refer to Table 1 for a comparison of PointNet++ and MeshNet. These two approaches make the following general assumptions about the data format:

1. An object *O* can be represented by a set of geometric primitives *O* = {*e*_*i*_}, i.e., the mesh vertices or faces. PointNet++ works with spatial point sets which are not necessarily from a mesh structure; we use the mesh representation to put the two methods under the same framework.
2. A geometric primitive *e* an be represented by a descriptor (or feature vector) *f*(*e*). There can be multiple such descriptors for each primitive, e.g., those generated by different layers of a deep neural network. The descriptor encodes information such as spatial location, appearance, and structural features.
3. The descriptor *f*(*e*) contains a subvector *f*_*s*_(*e*) called the spatial descriptor, which encodes the spatial location of *e*.
4. There is a well-defined topology on the set of geometric primitives. That is, for each geometric primitive *e* ∈ *O*, a neighborhood *N*(*e*) ⊂ *O* can be defined. This neighborhood associates *e* to a set of other primitives that are nearby or attached to *e*.
5. There is no natural order assumed for elements in *O*. For a neighborhood *N*(*e*), there might be (in the case of MeshNet) or might not be (in the case of PointNet + +) a natural order.

**Table 1:** Summarizes a comparison of PointNet++ and MeshNet.

	PointNet++	MeshNet
1. Geometric primitive	Vertices	Faces as triangles
2. Input descriptor	Position (× 3) + vertex normal (× 3)	Centers (× 3), corners (x3 × 3), face normals (× 3)
3. Descriptor	Position + MLP output channels	Symmetric combination of MLP over face-vertex feature pairs; MLP over face and neighbor normals
4. Neighborhood	Vertices within a fixed radius, or K-nearest neighbors if the number within radius exceeds K	Cross edge neighbors. Each face always has 3 neighbors
5. Order of neighborhood	Unordered	Defined by mesh. There can be a defined order (clockwise)
6. Feature Transformation subnet	MLP with batch normalization and ReLU	MLP with batch normalization and ReLU
7. Aggregation subnet	Max pooling of neighborhood;	Mesh Convolution, pooling and concatenation
9. Progressive spatial abstraction	Vertex cover of volume with progressively larger radius (named MSG in paper)	None

For the overall network architecture to mimic the deep convolutional structure of the CNN, a deep neural network built on top of such a sparse data representation typically specifies a recipe for each of the following aspects:

1. A feature transformation subnet, which converts a descriptor to a higher level of representation. This corresponds to 1 × 1 convolution which is typically used in a 2D CNN. This is typically realized with multiple linear layers (MLP) with activations like ReLU and optional drop-out and batch normalization.
2. An aggregation subnet, which converts *N*(*e*) into a single higher level of geometric primitive. This loosely corresponds to the convolution concept of a CNN. The aggregation subnet may generate a new geometric primitive *eʹ* or may preserve the spatial component of *e* and change only the rest of the descriptor. Both PointNet++ and MeshNet preserve the spatial properties of the geometric primitive instead of generating a new one.
3. A repeating composition of the above two structures that progressively converts the data to a representation of higher abstraction and/or lower resolution. MeshNet does not lower the resolution between blocks of layers, though receptive fields of neurons in the higher layers do enlarge because of information indirectly passing in through neighboring neurons.

### Data pipeline and implementation details

#### Mesh optimization

**Figure 2:**
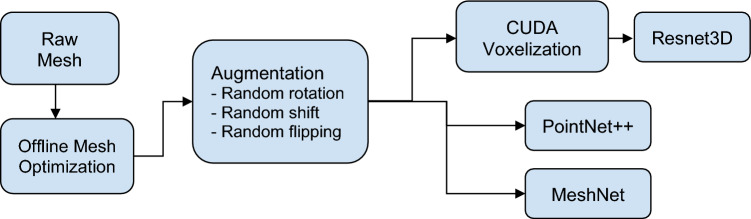
Data pipeline.

**Figure 3:**
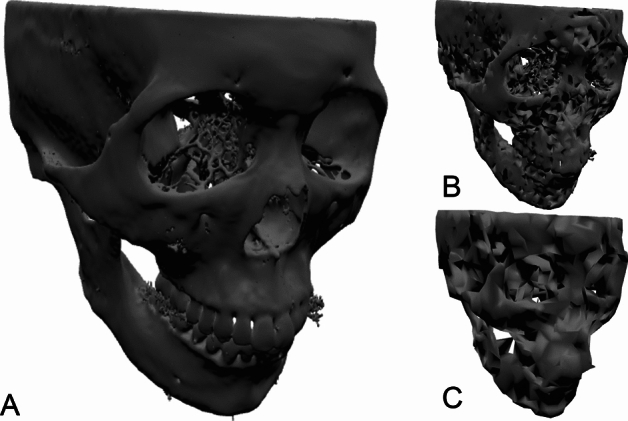
Rendering of a sample mesh. (**A**) Resnet3D resolution. (**B**) PointNet++ resolution. (**C**) MeshNet resolution.

Our data pipeline is illustrated in Fig. 2. Mesh data is offline optimized for size and rendering efficiency. The raw data is stored in the STL format and has a size ranging from 200 to 500 MB per sample. We applied offline mesh optimization (mainly vertex simplification) and generated three versions of mesh files. The different versions are optimized for each neural network architecture and are summarized in Table 2. Samples are shown in Fig. 3. The Resnet3D version is mainly constrained by file size to reduce disk loading time. As the mesh is voxelized into a 3D volume, the number of vertices does not affect the volume size. The Pointnet++ and MeshNet versions are constrained by GPU memory. All three methods are trained with a batch size of 8 on a GPU with 11 GB of memory. The meshes are optimized such that the maximal number of vertices or faces can be loaded into the GPU within such constraints. We see that the memory inefficiency of MeshNet gives it a disadvantage: the resolution of the mesh is substantially lower than the other two as shown in Fig. 3.

**Table 2:** Different versions of meshes used in the study.

Version	File size Mean ± sigma	Vertex count Mean ± sigma	Face count Mean ± sigma	Optimization target
Raw data	415 MB ± 99.7 MB	24.9 M ± 5.98 M	8.30 M ± 1.99 M	
A. Resnet3D	29.5 MB ± 7.10 MB	808 K ± 193 K	1652 K ± 398 K	Disk throughput
B. Pointnet++	795 KB ± 30.0 KB	18.6 K ± 2.0 K	47.6 K ± 0.7 K	GPU memory
C. MeshNet	117 KB ± 6.6 KB	2.21 K ± 0.3 K	7.52 K ± 0.3 K	GPU memory

#### Data augmentation with mesh

Data augmentation is crucial for the successful application of deep learning on relatively small datasets. For 3D data, the basic augmentation operations are random shifting and rotations, which can be implemented by the multiplication of a 3 × 3 transformation matrix and the 3D position vector. The augmentation is done in the mesh format, which affects only the vertices and is much more efficient than the augmentation of dense 3D volumes. We apply the following three augmentations:

- Random rotation within ± 10°
- Random shift within ± 5% of size, along each axis independently
- Random flipping (mirroring) at 50% chance

#### CUDA voxelization

Voxelization is the process to convert a mesh into a dense volume and is a relatively computationally intensive operation. We adopted a CUDA-based voxelization method that allows us to remove the CPU bottleneck and match the speed of neural network training.

#### Implementation details

The models were evaluated with a GeForce RTX 2080 Ti GPU with 11 GB memory. Table 3 provides a summary of the number of parameters of each architecture. The following software packages were integrated:

- Mesh Optimization: https://github.com/zeux/meshoptimizer
- Cuda Voxelizer: https://github.com/Forceflow/cuda_voxelizer
- Resnet3D: https://github.com/xmuyzz/3D-CNN-PyTorch
- PointNet++ (MSG): https://github.com/yanx27/Pointnet_Pointnet2_pytorch
- MeshNet: https://github.com/iMoonLab/MeshNet

**Table 3:** Summarizes the number of parameters of each architecture.

Architecture	Number of parameters
Resnet3D	63.5 M
PointNet++ (MSG)	1.7 M
MeshNet	4.2 M

### Method based on morphological gradients

A saliency map^9^ is typically applied to visualize the features of an input color image that contribute to classification decision making. There are, however, difficulties in applying saliency maps in our case. First, our input is a binary volume, so there is not much local texture as is found in color images and that contributes to decision making. Second, we are interested in visualizing the surface features rather than a difficult-to-visualize 3D volume of gradient values. In this section, we develop a new method based on morphological gradients, as a straightforward and intuitive way of visualizing surface features that influence decision making.

We represent a 3D model with a mesh, which is the triangulation of the 2D surface that encloses the 3D model. The morphological gradient is defined on any vertex of the mesh. The idea is to shift the location of the vertex along its norm and allow it to drag nearby vertices with it so that there is a local bump (or dent) in the 3D model. Then we compare the prediction values of the two meshes.

Let *M* be a 3D mesh and let *f* be a scoring function, in our case, the neural network model that maps a mesh to a binary classification score in the range of [0,1]. For any vertex *p*_0_ ∈ *M*, we can define a transformation function *T*(*p*;*p*_0_,*δ*) that slightly alters the location of any vertex *p* ∈ *M* along its norm *N*(*p*), according to how close *p* is to *p*_0_:

$$T(p;p_{0} ,\sigma ) = p + \delta \sigma \left( {\left\| {p - p_{0} } \right\|} \right)N\left( p \right),$$

where

- *δ* is the amount of change (A positive *δ* corresponds to a bump in the 3D model and a negative *δ* generates a dent) and
- *σ*(⋅) is a monotonically decreasing function of distance value such that *σ*(0) = 1 and $\mathop {\lim }\limits_{r \to + \infty } \sigma \left( r \right) = 0$.

We can similarly define *T*(*M*;* p*_0_, *δ*) to be the mesh with every vertex transformed by *T*. The morphological gradient at point *p*_0_ is therefore,

$$\nabla_{{p_{0} ;\delta }} f(M) = f\left[ {T\left( {M;p_{0} ,\sigma } \right)} \right] - f[M].$$

Note that the morphological gradient assigns a real value to any vertex in the mesh. This value can then be used to visualize a heat-map on the mesh surface.

Dense evaluation of morphological gradients is expensive as each evaluation costs a full model inference. We assume the continuity of the gradient and apply a sparsification of the mesh with vertex point cover. A vertex point cover *C*(*M*;*ρ*) of a mesh *M* is a subset of *M*’s vertices such that for any *p* ∈ *M* there exists at least one *pʹ* ∈ *C*(*M*;*ρ*) such that the geodesic distance $\left\| {p - p^{\prime} } \right\| < \rho$. The point cover can be found by breadth-first traversal of *M* as a graph, with the geodesic distance approximated by distance on the graph. After we obtain the gradient values on the point cover, we then interpolate them for values on all mesh vertices.

In our implementation, we want to define the transformation function *T* to model the actual alterations that are possible by craniofacial surgery. That is, the bump (or dent) caused by *σ* should look natural. After exploring multiple alternatives, we found a linear function to be most visually appealing (as well as efficient to implement). With a range parameter *R*, we define the function to be,

$$\sigma \left( {r;R} \right) = \max \left\{ {0,\frac{R - r}{R}} \right\}.$$

### Ethics

This study was approved by the Cedars-Sinai Medical Center Institutional Review Board (IRB).

## Results

### Cross validation results

For model evaluation, we employed stratified five-fold cross-validation. Here, the entire dataset is split into five equal parts, maintaining the class distribution. Each part (20%) serves as the testing set once, while the other four parts (80%) are used for training. This process is repeated five times, with results recorded each time. As the dataset is small, we compared the average validation AUC versus training epoch curves of different methods, where a better method is expected to have an overall higher curve after model convergence. This evaluation protocol was chosen for the following reasons:

- A more complete evaluation protocol would ask for separate validation and test phases. The hyperparameters of the model, especially the number of training epochs, are determined in the validation phase, and the optimal hyperparameters are applied to the test phase for comparison. However, with a small dataset size, it is not possible to allow the three-part splitting, as a training set that is too small would lead to substantial fluctuations in model performance (as can be seen in our results) and validation and testing sets that are too small would lead to unreliable determination of optimal hyperparameters and testing scores.
- With a small dataset size, comparing and reporting model accuracy using a single metric number would be unreliable. Therefore, we present our results qualitatively to show the overall accuracy of different methods.
- There are two typical metrics for binary classification problems: accuracy and AUC (or AUROC, area under the ROC curve). Accuracy is a more straightforward metric, but it requires us to determine a threshold that converts a 0–1 belief score into two classes. We observed that our model predictions have an overall fluctuating bias towards 0 or 1, and the midpoint is rarely close to 0.5. Using a fixed threshold would generate a relatively low accuracy score even when AUC is above 0.9. Such accuracy metrics would be similarly low and fluctuating for different methods, making it difficult to compare them.

**Figure 4:**
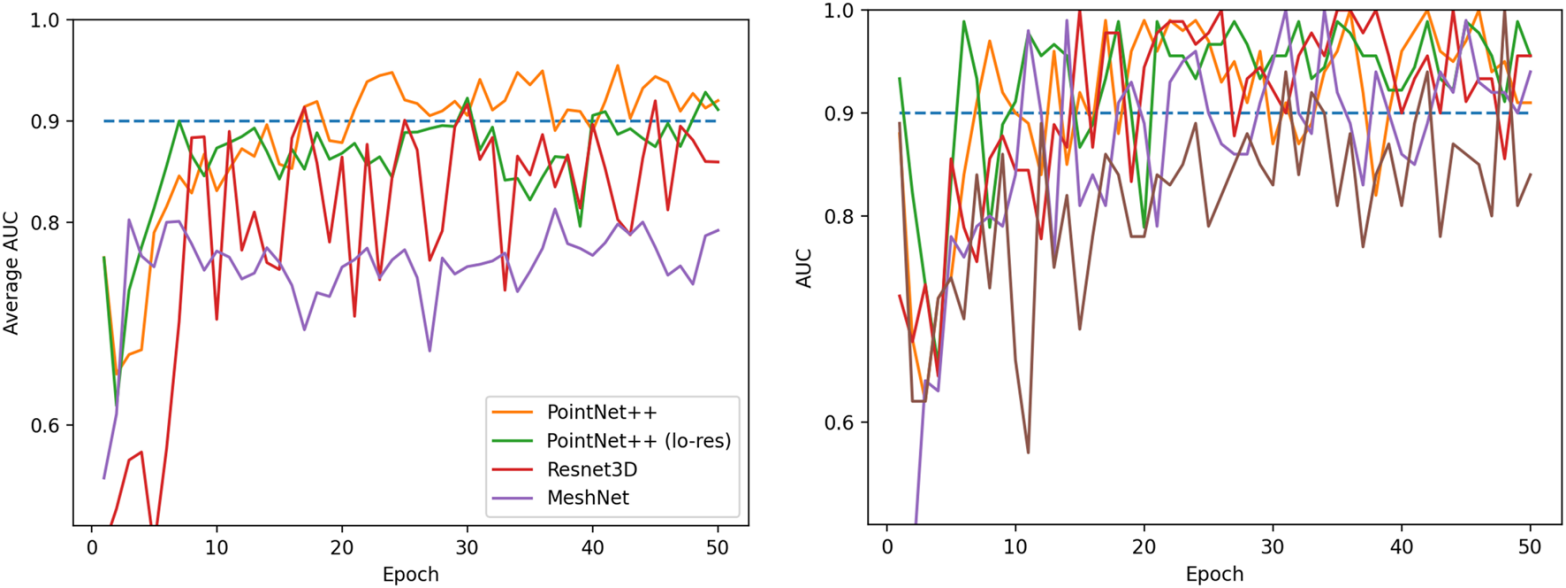
(Left) Cross validation AUC (average across five folds) of different methods with the same batch size and memory constraint. (Right) AUC of PointNet++, all five folds.

Our primary comparison results are shown in Fig. 4. We see that PointNet++ outperforms the other methods. The accuracy of Resnet3D lies in between, but the fluctuation of the curve is very large. We believe this is mainly because the number of parameters of Resnet3D is substantially larger than the other two methods, and it is therefore more difficult to reach a stable convergence with a limited number of training examples.

Because the training examples for MeshNet were reduced to a resolution that is much lower than those used for the other two methods, there are two possible explanations of the low accuracy of MeshNet: either the method itself does not work as well on the skull dataset or the mesh resolution is too low. To address this uncertainty, we evaluated PointNet++ on the same low resolution meshes used to train MeshNet (the lo-res curve of PointNet + +). We see that even with low resolution examples, the AUC of PointNet++ is still significantly higher than MeshNet.

**Figure 5:**
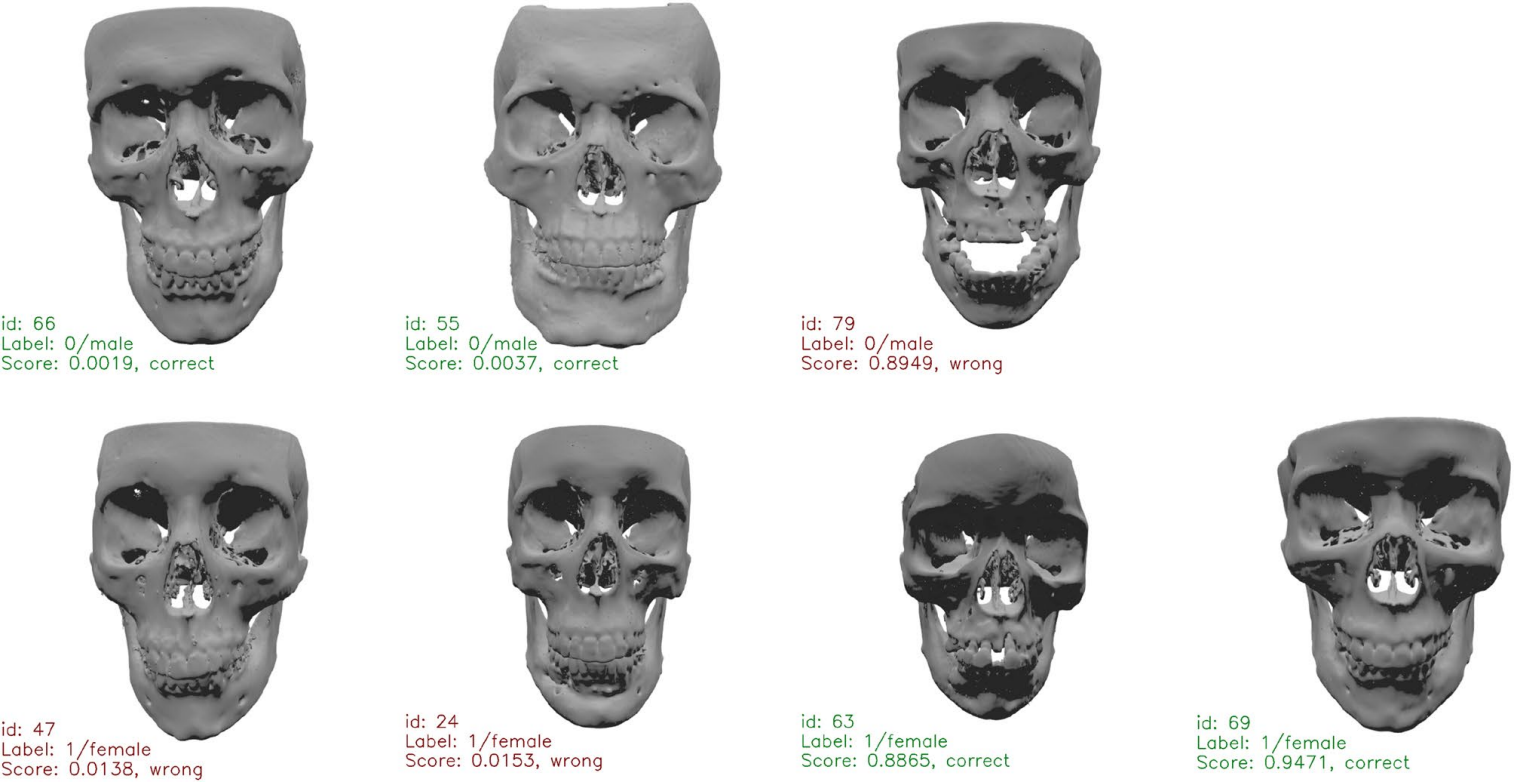
Examples of correct and incorrect classifications. Top row contains male samples (label = 0) and bottom row female (label = 1), each row is ordered by prediction score. There are more misclassified female samples and only one misclassified male sample.

The right side of Fig. 4 shows the individual curves of the five-fold experiments. We observe a very large spread of AUC across different folds. The three higher curves are mostly above 0.9 after convergence and before overfitting starts after the 40th epoch. The worst curve, however, intermittently drops below 0.8. We believe this is mainly due to the small number (20) of testing examples. A few difficult examples can cause a relatively big drop in test AUC. Figure 5 shows some of the correctly and incorrectly classified test samples from the worst epoch (Epoch 36) of the lowest PointNet++ curve (Fold 0). Although institutional imaging protocols mandate that the mouth be closed, either due to non-compliance, operator negligence or pathology, some subjects that were included in the study exhibited mandible malposition, which may have affected the algorithm.

There are several possible reasons that some skulls may be misclassified in terms of sex. One reason, as illustrated in Fig. 1, is the significant overlap in the bimodal quantifiable measurements for presumed sexually dimorphic traits. A second contributing factor is the contribution of genetic factors that may be considered prevalent in a given population group^10^. In other words, among one group, a given trait may be less dimorphic than another, or phenotypes may present differently between the sexes than in other groups^11^. For example, facial dimensions such as skull radius vary by height, and average height varies among different racial groups^12^. An individual with small stature that is average for one population might be considered well below average height in another group^13^. Race is a societal construct, not easily defined, because it is influenced by an individual’s self-reported identity and heterogeneous ancestry. Thus, controlling for this factor even with a large data set can be challenging, if not impossible. A third confounding variable is age, which also contributes to variations in skull shape and sexually dimorphic traits during development and, to a more subtle degree, through adulthood^11,14^. Lastly, as discussed later, asymmetry (the right side being more prominent) is a more masculine trait according to some investigators and this may have some effect on the algorithm’s bias in judging skull shape^15^. If this is true, greater symmetry may be interpreted as more feminine.

Note that the CT scans for this study were partial scans of the head that did not encompass the whole head. The scans were presenting the model with a view that included the truncated braincase. And the extent of the truncation does vary somewhat across the scans. Given this variation, the truncations should be seen as a source of noise within the dataset. A future study could add a training augmentation in which the braincase truncation is varied (by cropping a small random amount from each training image), thus making the model more robust against these variations.

### Visualization with morphological gradients

**Figure 6:**
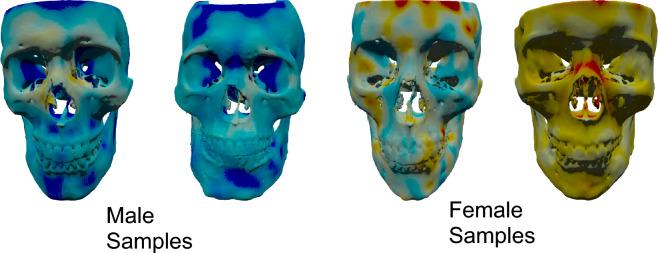
Visualization of morphological gradients. Red means that pushing the area outward makes the skull more female-like while blue means that pushing the area outward makes the skull more male-like.

Figure 6 shows samples of morphological gradients calculated using the Resnet3D model.

Historical analyses of human adult facial sexual dimorphism measured in various ways have revealed several attributes (both soft tissue and bony) that we expect to find as more masculine or feminine^10,13,16^. The female face tends to have a more overall square shape (as measured by the ratio of the horizontal and vertical dimensions) while the male face is typically rectangular (greater in the vertical dimension according to Ferrario et al.^15^). Vertical distances on the face, in general are longer in males. We think the mesh models may not capture these ratios (width/height) very well because they are better at measuring topography.

In the upper face, a lower and more prominent brow ridge is a masculine trait, as reflected in three of the gradients (flatter brow ridges are more blue indicating that if “pushed out” these features would make the subject more masculine)^14^. In the lower face, the mandible is relatively wider and the chin (pogonion) is lower in masculine skulls, a finding also supported by three of the gradients shown^15^. Some authors have reported that the masculine face exhibits greater directional asymmetry than the female face, with the right side typically longer or more prominent^17–21^. Conversely, the left side of the male face tends to exhibit less dimorphism when compared with a typical female face. In one of the female gradients, the subject’s right side has more blue color, suggesting that greater prominence would enhance masculinity.

The heat-map visualization of different skulls brings to light possible directions for future improvement. First, the gradient visualization shows less mirror symmetry than what is present in the skull model. This is likely because either the model is not completely symmetric due to the imperfectness of training data or this truly represents a dimorphic trait. One way to improve the model symmetry would be to evaluate the gradients using a mirrored sample and then take the average. It will be very interesting if the asymmetry is truly a dimorphic trait; this will need more extensive study to confirm, including testing with a larger training and validation dataset. Second, currently the gradients are evaluated at all locations including the non-facial internal regions of the skulls. This makes the computation process unnecessarily long and generates colorization in distracting areas. These potential improvements require mesh registration and segmentation methods which would allow us to standardize the pose of the 3D model and restrain processing within specified regions.

## Related work

The earliest studies of human sexual dimorphism were performed by directly measuring dimensions and distances between anatomic landmarks on human skulls. This practice was important to paleoanthropologists, forensic pathologists and anatomists for the study and identification of bones from humans of unknown age, sex and racial group^22^. With the introduction of x-ray radiography, standardized views (cephalograms) allowed for more standardized measurements to aid in the study, diagnosis and management of trauma, orthognathic conditions and congenital deformity affecting the facial skeleton^23^. 3D computed tomography brought even more power to the study of anatomy, opening the door to reconstruction of images into a more intuitive 3D framework that can differentiate soft tissue from bone and that allows for more reproducible measurements. CT scans are now routinely employed in planning facial and skull surgery, including reconstruction^24^. Using virtual surgery planning, the reconstructive surgeon can order cutting guides and prefabricated plates to replace or restore missing portions of the anatomy.

Today, a combination of these technologies is used to describe sexual dimorphism of the human face and facial skeleton^25^. Several studies in the medical literature focus on surface morphology, since appearance has an effect on subjective attractiveness^10,26^. The facial skeleton has implications on both appearance and function, though it has gained less attention when discussing sexual dimorphism and implications for attractiveness^12^.

Bannister et al.^27^ applied 3D facial surface image analysis to quantify the effect of sex on adult facial size. Such objective metrics provide a guideline for gender-affirming facial surgery. By performing a masculinity-femininity analysis on any given facial skeleton, an individualized plan to achieve a particular goal (such as facial feminization) can be formulated.

Li et al.^28^ introduced a large scale public dataset of 3D meshes (MedShapeNet), including skull meshes derived from CT and MRI images, along with meshes for other bones, organs, vessels, and muscles.

The main application of deep neural networks on skull data is automatic virtual skull reconstruction^29–32^. Patient-specific cranial implants are currently designed by experts and are expensive. The symmetry can be exploited in some cases to accelerate the process, but such methods fail with midspan defects or bilateral defects that occur in both sides of the skull. Unlike the classification task evaluated in this paper, reconstruction makes use of the image/volume generation capability of deep neural networks. The previous works were all based on dense volume/slice representation. The superiority of PointNet++ demonstrated in this paper suggests that mesh could potentially be a better representation for reconstruction tasks as well. However, the encoder-decoder architecture of the volume-generating networks cannot be easily adopted for mesh representation, and how to design a mesh-generating network or how to generate volumes with mesh inputs remains a challenge to be solved in the future. Before deep learning, statistical shape modeling was the primary method used in skull reconstruction^33,34^.

## Conclusion

In this study, we retrospectively collected a dataset of 98 3D skull images which is the first of its kind for 3D medical imaging study, and we conducted an evaluation of three deep neural network architectures (Resnet3D, PointNet++, and MeshNet) for the sex classification of 3D skull images. Our results indicate that PointNet++ achieved the best overall accuracy as measured by AUC. Notably, we observed AUC values above 0.9 with a small training set of only 78 examples, and we anticipate that useful models could be trained with a larger training set of hundreds of samples, or by including an auxiliary dataset such as MedShapeNet^28^ to augment training. Additionally, we developed a new method based on morphological gradients, which demonstrates how local skull shape alterations can make the skull more male- or female-like. This visualization technique suggests that the neural network models could potentially assist in planning gender-affirming craniofacial surgery. Overall, our study highlights the potential of deep neural network architectures for sex classification of 3D skull images and offers insights into potential clinical applications.

## Data Availability

The clinical data and 3D skull image data for this study were collected and prepared by the researchers of Cedars-Siani Medical Center. As data collection is on-going for project stage-I, raw data is not currently available for data sharing to the public. The datasets used and/or analyzed during the current study are available from the corresponding author on reasonable request.
